# Distinguishing granulomatous lobular mastitis from breast cancer using a clinical–radiomics nomogram to improve diagnostic accuracy

**DOI:** 10.3389/fonc.2026.1720213

**Published:** 2026-03-11

**Authors:** Yue Wang, Ximeng Zuo, Junxiu Zhai, Xiang Gao, Zhenzhou Chen, Xiaofa Li, Weiqi Pei, Xiaoguang Shi

**Affiliations:** 1 Department of Breast Surgery, Dongzhimen Hospital Affiliated with Beijing University of Chinese Medicine, Beijing, China; 2 Department of Ultrasound, Dongzhimen Hospital Affiliated with Beijing University of Chinese Medicine, Beijing, China

**Keywords:** breast cancer, diagnosis, granulomatous mastitis, hematologic tests, medical records, radiomics

## Abstract

**Background:**

Granulomatous lobular mastitis (GLM) is often misdiagnosed clinically as breast cancer (BC). Therefore, it is crucial to differentiate between GLM and BC.

**Methods:**

This study used 259 samples from 129 patients with GLM and 130 with BC. A total of 874 radiomics and 11 clinical features were obtained. The least absolute shrinkage and selection operator algorithm was used to select radiomics features. Univariate and multivariate analyses were performed to screen clinical features. Three machine learning algorithms were applied to assess the efficiency of the radiomics, clinical, and combined models, which were compared to select the optimal model. Finally, a nomogram based on the optimal model was developed. Decision curve analysis (DCA) and calibration curves were used to assess the clinical utility of the nomogram.

**Results:**

Twelve radiomics features were identified as the most relevant for distinguishing GLM from BC, including the original gray level co-occurrence matrix autocorrelation feature. Important clinical features included age, nipple inversion, and C-reactive protein levels. The combined model demonstrated superior performance in terms of accuracy, specificity, and sensitivity compared with the clinical and radiomics models. A nomogram was constructed based on the combined model. The calibration curve and DCA further confirmed the superior clinical value of the nomogram.

**Conclusion:**

A combined model incorporating 12 radiomics and 3 clinical features is potentially valuable for distinguishing GLM from BC.

## 1 Introduction

Granulomatous lobular mastitis (GLM) is a benign inflammatory disease of the breast, primarily observed in nonlactating women of reproductive age with a history of pregnancy and breastfeeding (1).

This disease is closely associated with race and region. In recent years, the number of cases reported in Asian and Mediterranean countries, including China, Iran, and Turkey, has substantially increased. Currently, the etiology of GLM remains unclear (2). Lactation disorders caused by milk stasis, hyperprolactinemia, and breast trauma are considered potential predisposing factors for the disease (1). Breast cancer (BC) is the second most common malignant tumor and a leading cause of cancer-related mortality among women, making it a major focus of women’s health because of its high incidence and mortality (3). Epidemiological surveys have shown that the number of new cases of BC in women increased to 1,977,212 globally in 2019 (4), and the number of new BC cases is predicted to exceed 3 million annually by 2040 (5).BC and GLM have been reported to be highly similar in clinical and imaging findings (6). Clinical experience indicates that because of the disease characteristics of GLM, it is frequently misdiagnosed as BC. This may lead to inappropriate treatment and impose a substantial psychological burden on patients. Therefore, distinguishing between GLM and BC is crucial in clinical diagnosis and treatment.

Radiomics is an imaging analysis approach that is applied across multiple biomedical fields to distinguish benign from malignant tumors, predict disease course and survival, and evaluate treatment response (7, 8). Growing evidence indicates that medical images often contain more information than can be seen with the naked eye and that the extracted image parameters have a certain correlation with the clinical characteristics of the disease (9). Previous studies have used contrast-enhanced ultrasound (CEUS) to characterize imaging features and distinguish GLM from BC, demonstrating good discriminatory performance. Moreover, the use of CEUS in clinical practice can influence decisions regarding further biopsy (10).These findings provide an important basis for the present study to perform differential diagnosis using conventional ultrasound images combined with radiomics techniques.

Based on clinical and radiomics data, this study identified clinical factors and imaging features associated with GLM and BC, employed machine learning to develop and select the optimal model, and subsequently constructed a nomogram using this model. Decision curve analysis (DCA) was then performed to evaluate the clinical utility of the model, thereby providing a novel clinical–radiomics approach for distinguishing GLM from BC.

## 2 Materials and methods

### 2.1 Study participants

The patient inclusion criteria were as follows: (i) availability of complete examination data; (ii) availability of surgical or biopsy results; (iii) no history of prior breast surgery, biopsy, chemotherapy, or radiotherapy; and (iv) evaluation of the most suspicious lesion in patients with multiple lesions. Finally, 259 samples were obtained, including 129 patients with GLM and 130 with BC. All patients were randomly assigned to training and validation cohorts in an 8:2 ratio using the “random” function in Python (version 3.7.3). Subsequently, 80% of the GLM samples and 80% of the BC samples were combined to form the training set, whereas the remaining 20% of the GLM samples and 20% of the BC samples were used as the validation set.

This study was conducted in accordance with the principles of the Declaration of Helsinki and obtained approval from the Ethics Committee of Dongzhimen Hospital Affiliated to Beijing University of Chinese Medicine (approval no: 2025DZMEC-437-03).

### 2.2 Clinical, laboratory, and radiomics features

Routine laboratory tests are commonly performed in clinical practice to evaluate breast lesions and may have potential diagnostic value for distinguishing GLM from BC. To capture these potentially informative markers, 11 nonimaging features were included in the analysis, comprising (i) clinical characteristics obtained from history and physical examination such as age, smoking history, and nipple inversion and (ii) laboratory parameters obtained from blood tests such as white blood cell count, absolute neutrophil count, absolute lymphocyte count, absolute eosinophil count, absolute basophil count, C-reactive protein (CRP) level, prolactin level, and interleukin (IL) level.

To delineate regions of interest (ROIs) for radiomics feature extraction of GLM and BC, all images were normalized using the N4ITK bias correction algorithm to standardize image intensity distributions. Meanwhile, the 3D Slicer software (https://www.slicer.org/) was used to semiautomatically segment the three-dimensional ROIs for GLM and BC. The features of ultrasound images of patients with GLM and those with BC were extracted using the “pyradiomics” package in Python, and 1,023 image features were obtained.

To evaluate inter- and intraobserver agreement in radiomics feature extraction, ultrasound images from 24 patients were randomly selected. Two experienced sonographers independently delineated the ROIs following the same protocol, and the process was repeated after 2 weeks. Intraclass correlation coefficients (ICC) were then calculated, and GLM and BC radiomics features with ICC > 0.75 were retained for subsequent analysis.

Ultrasound images were acquired using two ultrasound devices, SUPERSONIC IMAGINE Aixplorer V and PHILIPS EPIQ Elite, both equipped with a high-frequency linear-array transducer (10–15 MHz) capable of real-time shear-wave elastography. Although the key imaging parameters (e.g., depth and gain) were adjusted for each patient to achieve optimal diagnostic quality, all adjustments were performed by experienced sonographers following the manufacturer’s recommended preset for breast scanning. This clinical practice, combined with subsequent bias correction and image standardization, aimed to minimize nonbiological variance in radiomics feature extraction.

### 2.3 Important feature screening

For clinical features, univariate and multivariate analyses were performed using the “dplyr” (version 1.1.4) (11) and “tidyr” (version 1.3.1) (12) packages to identify important clinical features associated with GLM and BC (p < 0.05). An odds ratio (OR) greater than 1 in the multivariate regression indicated a positive correlation between the clinical factor and the outcome event (BC). This factor would increase the occurrence risk of the outcome event. An OR less than 1 indicated a negative correlation between the factor and the outcome event, which would reduce the occurrence risk of the outcome event.

Radiomics features were normalized using z-score normalization across all extracted features. To eliminate redundant features, initial dimensionality reduction was performed using the SelectKbest method, followed by feature selection with the least absolute shrinkage and selection operator (LASSO) algorithm implemented via “scikit-learn” package in Python. We analyzed the radiomics feature coefficients retained by the LASSO model. A positive feature coefficient represented a positive correlation between the feature and the target variable (BC) being studied, indicating that higher feature values corresponded to a greater likelihood or extent of the occurrence of the target variable. In contrast, a negative coefficient represented a negative correlation between the feature and the target variable, indicating that the likelihood of the occurrence of the target variable decreases as the value of this feature increases. The greater the absolute value of a feature’s coefficient, the stronger its effect on the target variable and the more prominent its role in the model.

### 2.4 Model construction

We employed three algorithms, logistic regression (LR), multilayer perceptron (MLP), and support vector machine (SVM), to develop three models: a clinical model based on significant clinical features, a radiomics model based on significant radiomics features, and a combined model incorporating both feature sets. All input features were standardized by removing the mean and scaling to unit variance before model construction. Model performance was evaluated using five-fold cross-validation. For each fold, the receiver operating characteristic curve was plotted and the area under the curve (AUC) was calculated, with AUC > 0.7 indicating good performance. Sensitivity (TP/[TP+FN]) and specificity (TP/[TP+FP]) were also computed, where TP, FP, TN, and FN represent true positives, false positives, true negatives, and false negatives, respectively. The model with the best overall metrics was selected as the final model.

Based on the optimal model obtained, a nomogram was constructed. To assess the predictive performance of the nomogram, the calibration curve was plotted using the “rms” (version 6.8.1) (13) package. Among them, the radiomics features were represented by the radiomics score (Rad-score). The specific formula is as follows: Rad-score = β0 + β1F1 + β2F2 + … + βn*Fn, where F denotes the feature value, β denotes the coefficient of feature F, and n denotes the serial number of the feature. The Hosmer–Lemeshow (HL) test assesses model fit by evaluating the discrepancy between predicted and observed values. A p-value greater than 0.05 indicated no significant difference between the predicted and observed values. Additionally, DCA was performed using the “rmda” package (version 1.6) (14).

### 2.5 Statistical analysis

Statistical analyses were performed using Python (version 3.7.3) and R (version 4.2.2). Independent samples *t*-test was used for continuous variables that conformed to the normal distribution. Mann-Whitney *U* test was used for variables that did not conform to the normal distribution. The chi-square test was used to analyze discrete variables. A p-value of <0.05 was considered statistically significant for all statistical tests.

## 3 Results

### 3.1 Identification of important radiomics features and clinical features

**Figure 1:**
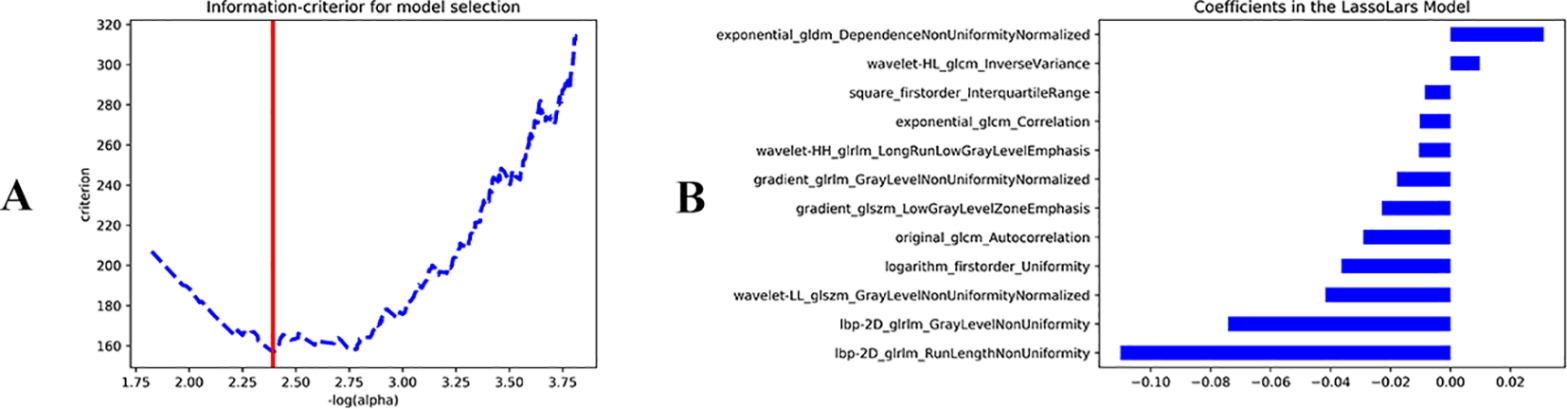
Least absolute shrinkage and selection operator **(LASSO)** analysis. **(A)** LassoLar. **(B)** Lassolars_22_coeff.

The original data yielded 1,023 radiomics features (**Supplementary Table 1**). A total of 874 radiomics features with ICC values greater than 0.75 were retained for analysis (**Supplementary Table 2**). LASSO analysis identified 12 important radiomics features, including original gray level co-occurrence matrix (GLCM) autocorrelation, exponential GLCM correlation, and logarithm first-order uniformity (**Figure 1**; **Table 1**). The coefficients of exponential_gldm_DependenceNonUniformityNormalized and wavelet-HL_glcm_InverseVariance were positive, indicating that higher values of these features are associated with an increased likelihood of BC. The remaining 10 features were negatively correlated with BC occurrence, indicating that higher values of these features corresponded to a lower likelihood of BC occurrence. Moreover, the feature lbp-2D_glrlm_RunLengthNonUniformity showed a stronger effect and may serve as a particularly important reference in clinical diagnosis.

**Table 1:** Key ultrasound radiomics features.

Features	Features coefficient
Original_glcm_Autocorrelation	-0.029
Exponential_glcm_Correlation	-0.0102
Exponential_gldm_DependenceNonUniformityNormalized	0.0311
Gradient_glrlm_GrayLevelNonUniformityNormalized	-0.0179
Gradient_glszm_LowGrayLevelZoneEmphasis	-0.0229
Lbp-2D_glrlm_GrayLevelNonUniformity	-0.0741
Lbp-2D_glrlm_RunLengthNonUniformity	-0.1101
Logarithm_firstorder_Uniformity	-0.0364
Square_firstorder_InterquartileRange	-0.0085
Wavelet-HL_glcm_InverseVariance	0.0098
Wavelet-HH_glrlm_LongRunLowGrayLevelEmphasis	-0.0105
Wavelet-LL_glszm_GrayLevelNonUniformityNormalized	-0.0417

Based on univariate (*p* < 0.001) and multivariate regression (*p* < 0.05), age (OR = 1.357), nipple inversion (OR = 0.266), and CRP level (OR = 0.768) were identified as the final important clinical characteristics (*p* < 0.001) (**Tables 2**, **3**). Based on the findings, age showed a positive association with BC occurrence, whereas nipple inversion and CRP levels were negatively correlated with BC occurrence.

**Table 2:** Final important clinical characteristics (univariate regression).

Feature	*P*-value
Age	<0.001
Smoking history	<0.001
Nipple inversion	<0.001
White blood cell count	<0.001
Absolute neutrophil count	<0.001
Absolute lymphocyte count	0.031
Absolute lymphocyte count	0.177
Absolute basophil count	0.253
C-reactive protein level	<0.001
Prolactin level	<0.001
Interleukin level	<0.001

**Table 3:** Final important clinical characteristics (multivariate regression).

Feature	OR	95%CI	*P*-value
Age	1.357	(0.001-0.009)	<0.001
Smoking history	0.092	(1.237-1.528)	0.14
Nipple inversion	0.266	(0.004-0.693)	0.036
White blood cell count	0.568	(0.247-2.522)	0.318
Absolute neutrophil count	1.154	(0.243-2.817)	0.811
Absolute lymphocyte count	2.152	(0.262-0.950)	0.375
C-reactive protein level	0.768	(0.981-1.068)	0.021
Prolactin level	1.024	(0.910-1.083)	0.27
Interleukin level	0.986	(0.071-0.881)	0.726

### 3.2 The combined model had better prediction performance

**Figure 2:**
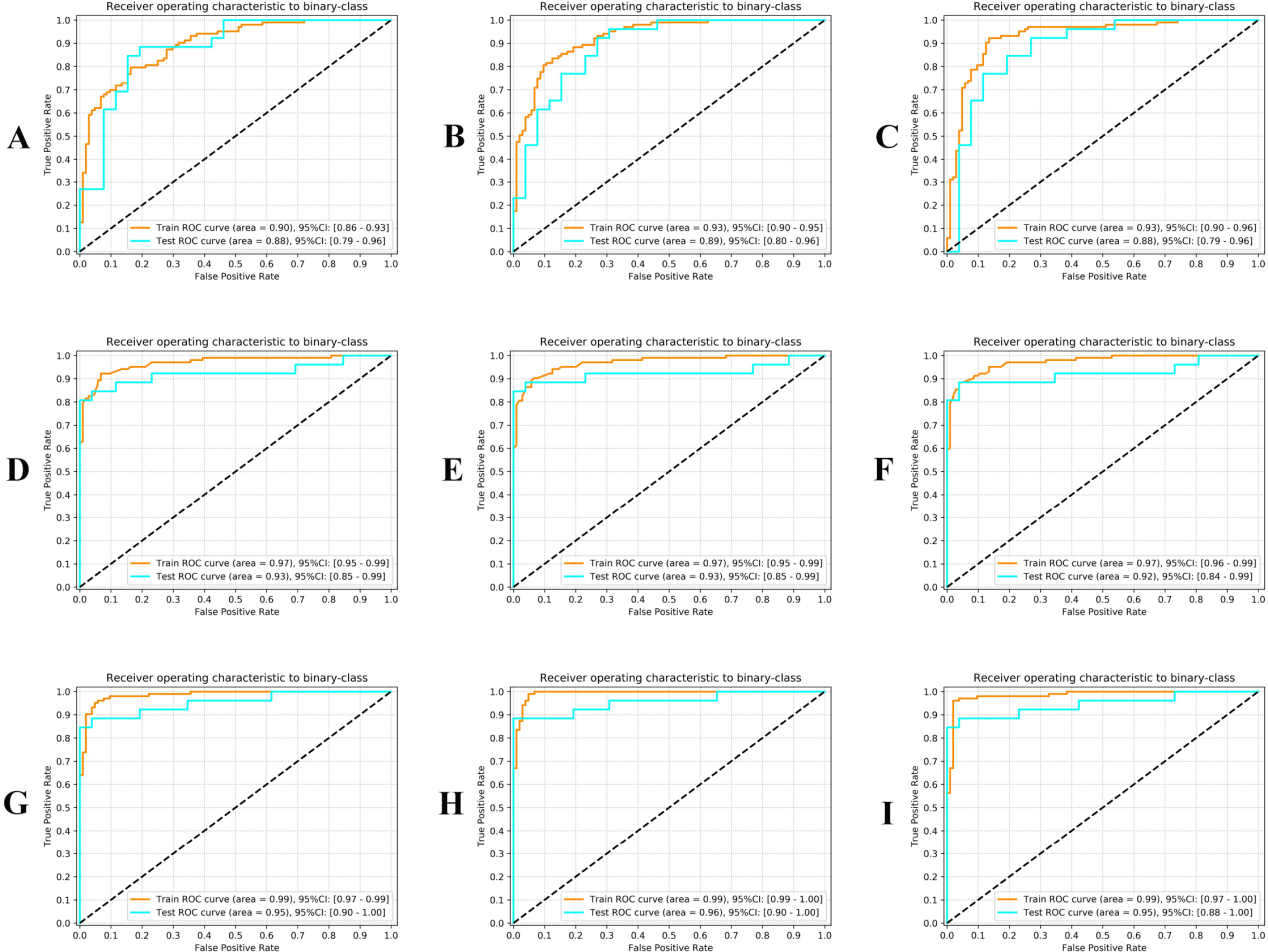
Model performance. **(A-C)** Performance of radiomics models developed using LP, MLP, and SVM. **(D-F)** Performance of clinical models. **(G-I)** Performance of the combined model.

**Table 4** and **Figures 2A–C** show the performance of the radiomics model developed using the LPR, MLP, and SVM methods. **Table 5** and **Figures 2D–F** show the performance of clinical models. **Table 6** and **Figures 2G–I** show the performance of the combined models. The combined models demonstrated higher sensitivity (LR: 0.96 and 0.88, SVM: 0.96 and 0.88, MLP: 0.99 and 0.88), specificity (LR: 0.94 and 0.96, SVM: 0.98 and 0.96, MLP: 0.95 and 0.92), and AUC (LR: AUC = 0.99 and 0.95, SVM: AUC = 0.99 and 0.95, MLP: AUC = 0.99 and 0.96) in both training and validation sets.

**Table 4:** Performance of radiomics models developed using LP, MLP, and SVM methods.

Model	Sensitivity		Specificity		AUC	
	Training set	Validation set	Training set	Validation set	Training set	Validation set
SVM	0.92	0.85	0.87	0.81	0.93	0.88
LR	0.8	0.77	0.84	0.85	0.9	0.88
MLP	0.83	0.77	0.88	0.81	0.93	0.89

**Table 5:** Performance of clinical models.

Model	Sensitivity		Specificity		AUC	
	Training set	Validation set	Training set	Validation set	Training set	Validation set
SVM	0.88	0.81	0.96	1	0.97	0.92
LR	0.92	0.81	0.93	1	0.97	0.93
MLP	0.9	0.81	0.93	1	0.97	0.93

**Table 6:** Performance of the combined model.

Model	Sensitivity		Specificity		AUC	
	Training set	Validation set	Training set	Validation set	Training set	Validation set
SVM	0.96	0.88	0.98	0.96	0.99	0.95
LR	0.96	0.88	0.94	0.96	0.99	0.95
MLP	0.99	0.88	0.95	0.92	0.99	0.96

### 3.3 The nomogram had good efficacy

**Figure 3:**
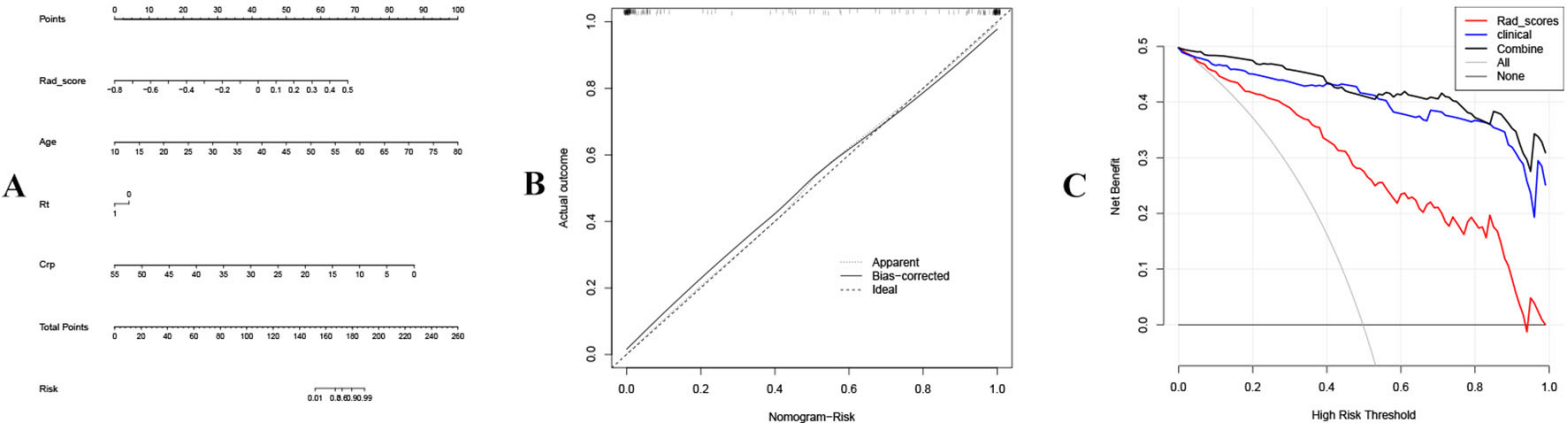
Nomogram and decision analysis results. **(A)** The nomogram. **(B)** Calibration curve of the nomogram. **(C)** Decision curve analysis results for the different models.

A nomogram was developed based on 12 important radiomics features and 3 important clinical features (**Figure 3A**). Each feature was assigned a score of one point, and the scores were summed to obtain the total points. The probability of BC occurrence also increased as the total points increased. The calibration curve of the nomogram nearly overlapped, indicating its high predictive accuracy (**Figure 3B**). The DCA revealed that the combined model was more beneficial than both the radiomics and clinical models. This was consistent with the above results (**Figure 3C**).

## Discussion

As a benign breast disease, GLM is highly similar to BC in terms of clinical manifestations and early imaging characteristics. The absence of specific distinguishing features increases the likelihood of misdiagnosis and poses substantial challenges to clinical differential diagnosis (15–17). However, radiomics can extract features associated with tumor pathophysiology and microenvironment, transform them into high-dimensional data, and show substantial potential for improving diagnosis, guiding treatment, and predicting prognosis in both diseases (18–20).

Based on clinical features and radiomics data, 3 important clinical features and 12 key radiomic features were successfully selected for analysis. GLM typically occurs in women of childbearing age, mostly between the ages of 30 and 40 years, especially within 5 years after pregnancy (1). The age distribution of BC is broader; however, it is more common in older women, with the incidence gradually increasing with age (21), which is consistent with the conclusion in section 3.1. Nipple inversion in patients with GLM often results from congenital ductal dysplasia and inherent lactation disorders. For example, flat or inverted nipples prevent infants from sucking, leading to poor milk drainage and subsequent disease (22). Notably, improper use of breast pumps may increase the risk of breast duct damage and increase the risk of developing GLM. Nipple inversion in BC is primarily caused by cancer cell infiltration of the nipple and surrounding tissues and is often accompanied by changes in breast skin, including peau d’orange appearance (23). This mechanistic difference provides a plausible explanation for our finding that nipple inversion was negatively correlated with BC (OR-0.266). Factors associated with GLM, such as congenital anomalies and ductal injury, are common in the GLM population, resulting in a higher prevalence of nipple inversion at presentation. In contrast, nipple inversion in BC usually occurs later, reflecting malignant invasion. Therefore, in our study cohort, this clinical feature is both a key morphological indicator and a distinct underlying pathophysiological process, thereby enhancing its value in distinguishing GLM from BC. Notably, this observed association is derived from a single-center cohort and may be subject to potential selection bias; thus, its generalizability requires further validation in multicenter studies. Nonetheless, this finding provides a practical diagnostic insight: when encountering patients presenting primarily with nipple inversion, after excluding congenital causes, clinicians should integrate this sign with other key clinical and imaging features, such as disease course and the presence of peau d’orange appearance, to refine the differential diagnosis between GLM and BC, thereby translating this statistical observation into improved clinical reasoning. Additionally, as an inflammatory disease, GLM often causes fever and increases CRP levels when local breast abscesses form (24). In patients with BC, CRP level may increase during disease progression in cases such as infection and tissue necrosis; however, it mostly remains unchanged in the early stage (25). In general, increased CRP levels in patients with GLM may be more significant, although this cannot be used as an absolute discriminative index. Therefore, combining this indicator with other imaging characteristics is crucial for improving differentiation accuracy.

The key radiomics features identified in this study capture the essential histopathological differences between GLM and BC. The feature “Exponential_gldm_DependenceNonUniformityNormalized” reflects the degree of nonuniformity in image texture dependence. A positive value of this feature indicates that the higher the feature value, the greater the tendency to diagnose the condition as BC. This is associated with the regular structure formed with the relatively uniform cell proliferation and fibroblast response within BC tumor tissue (26). In contrast, the heterogeneous tissue composition in GLM lesions caused by granulomatous inflammation, microabscess formation, and fat necrosis tends to produce lower values of this feature (27). Other texture-related features include Lbp-2D_glrlm_RunLengthNonUniformity and Lbp-2D_glrlm_GrayLevelNonUniformity. During the growth of BC tissue, relatively regular structures may form, conferring a degree of regularity and uniformity to the distribution of gray-level run lengths. In contrast, GLM masses demonstrate significant local texture variations in images due to factors such as inflammatory infiltration, tissue edema, and fat involvement (28–30). The feature “Wavelet-HL_glcm_InverseVariance” quantifies the smoothness of gray-level changes in the wavelet HL component, and its positive value is associated with BC diagnosis. Higher values may reflect relatively smooth and invasive tumor margins created by profibrotic responses and collagen deposition (31). In contrast, GLM lesions typically exhibit blurred boundaries due to inflammatory infiltration, and internal tissue necrosis, liquefaction, or inflammatory exudation produces irregular gray-level fluctuations in the HL component, resulting in lower feature values (32). These results are consistent with previous radiomics studies on distinguishing breast lesions and, for the first time, systematically quantify the differences between GLM and BC in texture regularity and edge/detail smoothness using conventional ultrasound images (33). Literature review showed that few studies have performed detailed analyses of GLM using these imaging indicators, highlighting the need to focus on such features in future GLM diagnosis.

A previous study developed a model based on ultrasound radiomics features to differentiate GLM from invasive breast cancer (IBC). The ultrasound radiomics model achieved optimal performance, with the AUC reaching 0.907 in the training set and 0.888 in the external validation set, indicating that radiomic features effectively improve the ability of ultrasound physicians to distinguish GLM from IBC. A nomogram combining ultrasound and radiomics features demonstrated substantial application prospects in noninvasive differentiation and diagnosis of GLM and IBC (34). In this study, clinical data were added to pure ultrasound images. The results showed that the composite model achieved higher AUCs (0.99 and 0.95 in the training and external validation sets, respectively) than both the clinical model and the radiomics-only model. This indicates that the composite model demonstrated significantly higher accuracy than the other two groups in the process of differentiating and diagnosing GLM and BC, thereby providing an effective noninvasive approach for clinicians to distinguish GLM from BC.

Although both GLM and BC present with poorly defined boundaries and invasive growth on conventional ultrasound images, radiomics features in this study revealed their key differences. The inflammatory infiltration of GLM manifests as high gray-level nonuniformity, which corresponds to tissue edema and microabscess formation (35). In contrast, neoplastic infiltration in BC shows clustered low-gray-level regions, reflecting dense cellular distribution within cancer nests (36). This difference originates from their distinct microenvironmental mechanisms, and ultimately, quantitative differentiation is achieved through the fusion model.

The tissue microenvironment provides the pathological basis for radiomics features (37). The tumor microenvironment (TME) is a special milieu that supports tumor survival, formed through interactions among tumor cells, various stromal cells, cytokines, chemokines, and other factors (38). Multiple cell types, blood and lymphatic networks, and the extracellular matrix (ECM) are key components of the TME and play a significant role in tumor infiltration (39). Adipocytes are dominant in breast tissue. Tumors influence surrounding adipocytes either through direct contact or via bioactive molecules, creating a tumor-associated adipocyte microenvironment (40). Bioactive adipokines released by adipocytes are major contributors to tumor progression (41). Leptin, an adipocyte-secreted hormone, promotes the secretion of ECM-remodeling enzymes matrix metallopeptidase 2(MMP-2) and matrix metallopeptidase 9(MMP-9) and induces invasion via focal adhesion kinase (FAK)-steroid receptor coactivator(Src)-dependent pathways, facilitating a more aggressive BC phenotype (42). Animal studies have shown that leptin interacts with ObR to activate the p38 and ERK signaling pathways, inducing IL-8 production in M2 macrophages, which increases tumor volume and mass, exacerbates lung metastasis, and reduces survival (43). Tumor-associated macrophages (TAMs) are another critical component of the breast TME. They are highly plastic in response to external signals and participate in innate and adaptive immune responses, thereby regulating multiple TME factors (44). TAMs play a key role in BC progression. They stimulate the process of tumor progression and induce resistance to various treatment types (45). Proline-rich tyrosine kinase 2(PYK2) mediates the crosstalk between BC cells and macrophages by exerting a critical effect on key receptor signaling. After PYK2 ablation in macrophages, the number of infiltrating tumor macrophages is significantly reduced, and tumor angiogenesis and growth are inhibited (46). Chemokines, through their interaction with specific receptors, recruit multiple subsets of immune cells and integrate them into the TME. These chemokines and their interactions have a substantial effect on tumor growth, progression, and treatment outcomes (47). The chemokine receptor axis C-C motif chemokine receptor 2(CCR2)-C-C motif chemokine 2(CCL2) plays a crucial role in the TME and strongly enhances the metastatic potential of BC cells (48). CCL2 is a poor prognostic marker in BC, enhancing the metastatic capacity of cancer cells and mediating stromal–tumor cell crosstalk in early-stage disease (49). The expression level of CCL2 in BC tissues has been reported to be much higher than that in healthy individuals (50). Moreover, CCR2 overexpression in BC can promote early disease progression via stroma-dependent CCL2 expression, providing important prognostic and therapeutic insights for ductal carcinoma *in situ* (DCIS) (51).

Currently, the etiology and pathogenesis of GLM remain unclear, and research on the microenvironment of GLM lesion tissues has attracted considerable attention. Single-cell RNA sequencing has revealed the immune landscape of this disease. The contents of immune cells, chemokines, and cytokines in GLM lesion tissues are significantly increased, with impaired estrogen response in breast luminal cells and improved angiogenic function of endothelial cells, compared with normal breast tissue. In terms of immune cells, the proportion of T cells and natural killer (NK) cells is increased, among which CD4+ T cells of all subsets and NK cells are significantly increased, and T helper 1 Th1 (Th1) cells account for a relatively high proportion of T cells and NK cells. In GLM lesion tissues, the gene expression levels of chemokines such as CCL3, CCL4, and C-X-C motif chemokine ligand 13(CXCL13) are significantly upregulated; meanwhile, macrophage phenotypes tend to differentiate into proinflammatory types, and pathways such as interferon (IFN)-α, IL-6/Janus kinase/signal transducer and activator of transcription 3, and tumor necrosis factor-α/nuclear factor kappa-light-chain-enhancer of activated B cells, are activated (52). The primary role of Th1 cells is to mediate cellular immunity and promote inflammatory responses, and IFN-γ, an important cytokine secreted by Th1 cells, has various immune functions. IFN-γ activates macrophages, enhancing their phagocytic and digestive capacity for foreign substances or cell debris, and upregulates MHC molecule expression, thereby strengthening the antigen-presenting function of antigen-presenting cells (53). Currently, no definitive research has focused on the specific role of the chemokine family in the occurrence and development of GLM. However, CXCL13 has been associated with some inflammatory and autoimmune diseases (54). Evidence indicates that adipocytes possess strong immune activity, and their immunity is regulated by interactions between adipocytes and various immune cells (55, 56). Adipocytes regulate immune cells in adipose tissue through lipid antigen presentation; however, the relationship between adipose tissue death and inflammation remains unclear. If apoptotic cells are not cleared rapidly and properly through endocytosis, their membranes may rupture, forming necrotic-like cells that further trigger inflammation (57). Adipocytes are an important component of the breast. A large number of immune cells and inflammatory factors accumulate locally when local breast inflammation occurs, thereby implicating adipocytes, disrupting their homeostatic environment, and inducing a series of reactions. IFN-γ derived from NK cells directly stimulates adipocytes, shifting their function from lipid synthesis to degradation and triggering lipid release (58). This finding is consistent with the characteristic of high gray-level fluctuations observed in the GLM images in this study. A study on pathogenic bacteria in GLM revealed that Corynebacterium parakroppenstedtii infection may cause GLM. Additionally, the study confirmed that this pathogenic bacterium is lipophilic, and fat-rich environments provide a more favorable condition for its infection (59). These findings warrant further in-depth analysis. In GLM, inflammation in the mammary lobules leads to local tissue congestion and edema, accompanied by an accumulation of immune cells and inflammatory cytokines that extend into surrounding adipose tissue. This promotes lipolysis, releasing lipids that act as antigens and continue to drive local inflammatory responses. Delayed clearance of affected or dead adipocytes further exacerbates inflammation, creating a vicious cycle that results in a combined inflammatory response within the mammary lobules, stroma, and adipose tissue. In patients with fatty-type breasts, the higher proportion of adipocytes may intensify the inflammatory response, leading to more extensive tissue damage. The local microenvironments in BC and GLM differ substantially, which provides a strong theoretical basis for the observed differences in radiomic characteristics of local masses in this study.

In this study, we constructed the first discriminative model integrating clinical parameters (age, nipple retraction, and CRP level) with radiomics features from conventional ultrasound. The model achieved an AUC of 0.95 in the validation set, outperforming single-modal models. However, several limitations of this study should be noted. First, this was a retrospective, single-center study, which may introduce selection bias and limit generalizability. Second, the model was evaluated internally and lacks independent external validation, which is crucial for confirming its robustness and clinical applicability. Third, imaging equipment availability and limited case numbers restricted data acquisition. Variations in image quality influenced by scanning parameters and patient cooperation may affect the accuracy and consistency of feature extraction. Future studies will expand the dataset through multicenter collaboration, standardize scanning protocols, and apply image preprocessing, including normalization and denoising, to improve image quality and data consistency. We also plan to refine breast classifications and perform detailed analyses of different types. Differences in data across institutions have hindered external validation, limiting clinical implementation. To address this, a standardized data-sharing platform can be established to facilitate collaboration and data exchange among institutions. The model will then be validated and optimized using multiple external datasets to improve its generalizability.

## Data Availability

The original contributions presented in the study are included in the article/**Supplementary Material**. Further inquiries can be directed to the corresponding author.
